# Trends in the use and nonmedical use of sedatives-hypnotics in the population aged 12 to 64 years in Taiwan: a comparative analysis of the national surveys in 2014 and 2018

**DOI:** 10.1186/s12889-024-20778-1

**Published:** 2024-11-25

**Authors:** Wei-Yi Tsay, Lian-Yu Chen, Shang-Chi Wu, Po-Chang Hsiao, Te-Tien Ting, Cheng-Fang Yen, Shu-Sen Chang, Chung-Yi Li, Hao-Jan Yang, Chia-Feng Yen, Chuan-Yu Chen, Jiun-Hau Huang, Yu-Kang Tu, Wei J. Chen

**Affiliations:** 1 Institute of Epidemiology and Preventive Medicine, College of Public Health, National Taiwan University, Taipei, Taiwan; 2 Department of Addiction Psychiatry and Kunming Prevention and Control Center, Taipei City Hospital, Taipei, Taiwan; 3 CTBC Center for Addiction Prevention and Policy Research, National Taiwan Normal University, Taipei, Taiwan; 4 School of Big Data Management, Soochow University, Taipei, Taiwan; 5 Department of Psychiatry, Graduate Institute of Medicine, College of Medicine, Kaohsiung Medical University Hospital & School of Medicine, Kaohsiung Medical University, Kaohsiung, Taiwan; 6 Department of Public Health, College of Public Health, National Taiwan University, Taipei, Taiwan; 7 Institute of Health Behaviors and Community Sciences, College of Public Health, National Taiwan University, Taipei, Taiwan; 8 Department of Public Health, College of Medicine, National Cheng Kung University, Tainan, Taiwan; 9 Department of Public Health, College of Health Care and Management, Chung Shan Medical University, Taichung, Taiwan; 10 Department of Public Health, College of Medicine, Tzu Chi University, Hualien, Taiwan; 11 Institute of Public Health, National Yang-Ming University, Taipei, Taiwan; 12 Center for Neuropsychiatric Research, National Health Research Institutes, Miaoli, Taiwan; 13 Department of Psychiatry, College of Medicine, National Taiwan University Hospital, National Taiwan University, Taipei, Taiwan

**Keywords:** Sedatives-hypnotics, Past-year use, Nonmedical use, Survey, Trends, Sex differences

## Abstract

**Background:**

Many countries, including Taiwan, have tightened regulations on prescribing sedatives-hypnotics over the concern of their associated adverse health effects. However, it remains seldom investigated whether different age-sex strata have differential trends in national surveys over time for either the use or nonmedical use (NMU) of sedatives-hypnotics. Comparing Taiwan’s two national surveys in 2014 and 2018, we aimed to examine (1) the population’s trends for the prevalence of past-year use and NMU of sedatives-hypnotics overall and in age-sex strata; (2) trends for sociodemographic subgroups for those age-sex strata with significant changes in past-year use and NMU of sedatives-hypnotics over time; and (3) trends for sources of and motives for NMU of sedatives-hypnotics.

**Methods:**

The national survey enrolled 17,837 participants in 2014 (response rate = 62.2%) and 18,626 participants in 2018 (response rate = 64.6%) of citizens aged 12–64 years. Each participant anonymously completed a computer-assisted self-interview. The questionnaire consisted of sociodemographic variables and the use of various psychoactive substances and sedative-hypnotics, among others. NMU of sedative-hypnotics was defined as using the drug without a prescription, or more frequently, or in larger doses than prescribed. To compare the prevalence between the two waves, we conducted multivariable logistic regression analysis and the difference-in-differences in prevalence was examined with an interaction term between survey year and sex.

**Results:**

We found decreasing trends in young adult (18–39 years old) males for both past-year use (3.07–2.29%) and NMU (0.84–0.18%), but increasing trends in adolescents (0.42–0.80%) and young adult females (2.91–3.81%) for past-year use and in adolescents (0.16–0.39%) and middle-aged adult (40–64 years old) females (0.73–1.14%) for past-year NMU of sedatives-hypnotics. Among the young and middle-aged adult females, the increasing trends for past-year use and NMU, respectively, were found to occur mainly in certain sociodemographic subgroups, with alcohol users being the overlapping subgroup.

**Conclusions:**

The differential trends over time of past-year use or NMU of sedatives-hypnotics in different age-sex strata in the population have policy implications to curtail the increasing trend over time.

**Supplementary Information:**

The online version contains supplementary material available at 10.1186/s12889-024-20778-1.

## Introduction

Sedatives-hypnotics are a class of prescription medications that enhance the inhibitory effects of γ-aminobutyric acid (GABA) to reduce tension or induce sleep [1]. Among them, benzodiazepines (BZDs), which bind to GABA receptors at the alpha 1, 2, 3, and 5 subtypes [2], have been approved for the treatment of anxiety and insomnia since the 1960s, and Z-drugs, which are structurally different from BZDs and selectively bind to the alpha 1 subunit [2], were introduced into the market later in the 1990s as safe alternatives for BZDs for the treatment of insomnia [3]. Since their introduction, the use of Z-drugs has been on the rise due to their quick onset, short half-life, and perception as being safer than BZDs [4, 5]. However, recent research has indicated that some Z-drugs are associated with an elevated risk of driving impairment or misuse [2, 6]. With an increasing trend of sedative-hypnotic prescriptions in populations such as adults in the U.S. from 1996 to 2013 [7], adults in Canada from 2001 to 2016 [8], and children, adolescents, and young adults in Sweden from 2006 to 2013 [9], concerns are increasing regarding their associated adverse health effects, including dependence and abuse problems, fatal overdose, traffic accidents due to decreased motor coordination [10, 11], falls and hip fractures among the elderly [12], and nonmedical use (NMU) [13]. Nevertheless, a recent study among adults in five Nordic countries from 2000 to 2020 revealed a decreased trend in the therapeutic intensity of BZDs and related drugs when use of sedative-hypnotics was substituted to other drugs with “sedative properties” such as quetiapine in non-psychotic doses [14].

Studies worldwide have reported that the prevalence of sedative-hypnotic use was higher among females than males and increased with age [15, 16]. One reason for the higher use prevalence among females is due to their higher prevalence of insomnia [17] and anxiety disorders [18] compared to males. Moreover, for people with anxiety disorders, females were more likely to show treatment-seeking behaviors than males [19]. In addition, the higher prevalence of sedative-hypnotic use among older adults than among younger adults might be due to the higher rates of insomnia and several comorbidities in older adults [20, 21], or a continuation of long-standing prescriptions in older adults. What remains seldom investigated is whether different age-sex strata have differential trends over time for either the use or NMU of sedatives-hypnotics.

Most research on trends for the use prevalence of sedatives-hypnotics has been based on claims data, where the prescription numbers or person-prescription prevalence of sedatives-hypnotics were estimated [7, 8, 20, 22, 23]. However, prescription patterns might not reflect the actual use of medications. In particular, claims data cannot provide estimates of NMU of sedatives-hypnotics, which has become a prominent form of psychoactive drug abuse in many countries, including the U.S [24, 25]. , the European Union [26], and Latin America [13]. One meta-analysis of 54 studies from 1996 to 2017 [27] found that common sources of NMU for sedatives-hypnotics were friends or family members [28]. Another systematic review of the motives for NMU of sedatives-hypnotics found that the most common motive was self-medication [29]. To date, however, the sources of and motives for NMU of sedatives-hypnotics in Asian populations remain seldom investigated.

After Taiwan implemented the National Health Insurance in 1995, people could relatively easily access prescription drugs with a low percentage of copayment at either a clinic or hospital. Despite the stringent regulations of sedatives-hypnotics as Schedule IV-controlled drugs since 1999 [30], three studies using claims data reported that the person-prescription prevalence of sedatives-hypnotics among persons aged 18 years old or older increased from 3.0% in 1997 to 7.3% in 2004 [31], the 1-year prevalence of sedatives-hypnotics use among persons aged 65 years old or older increased from 1.7% in 1997 to 5.5% in 2005 [32], and the number of person-days for prescription in the general population also increased from 4.0% in 2002 to 6.6% in 2009 [33]. Consistent with this trend, the yearly consumption of sedatives-hypnotics at the wholesale level reported to the Taiwan Food and Drug Administration increased from 2002 to 2010 [34]. Hence, new regulations on prescribing sedatives-hypnotics have been implemented since 2012, and any person who receives an abnormally excessive amount of sedatives-hypnotics in any clinical setting, including both hospitals and clinics, would be put on the watch-over list [35]. In addition, warnings over the risk of sleepwalking and sleep driving have been added to the package insert when prescribing zolpidem since 2013 [36]. Meanwhile, self-reported use and NMU of sedatives-hypnotics were explored for the first time in the 2014 National Survey of Substance Use in Taiwan. The past-year use prevalence of sedatives-hypnotics in 2014 was 5.46%, and the corresponding figure for NMU was 0.71% [37, 38], which was the first report of its kind in Asian populations. The impact of these new regulations on the prevalence of past-year use and NMU of sedatives-hypnotics over time in specific age-sex strata remains to be investigated. Furthermore, whether the sources of and motives for NMU of sedatives-hypnotics changed over time in Taiwan remains unknown.

To fill in these gaps in the literature, we turned to the 2014 and 2018 National Survey of Substance Use in Taiwan, which provide an opportunity to examine the trends for past-year use and NMU of sedatives-hypnotics over time at the individual level. This study aimed to examine (1) the population’s trends for the prevalence of past-year use and NMU of sedatives-hypnotics overall and in age-sex strata; (2) trends for sociodemographic subgroups for those age-sex strata with significant changes in past-year use and NMU of sedatives-hypnotics over time; and (3) trends for sources of and motives for NMU of sedatives-hypnotics.

## Methods

### Participants

The National Survey of Substance Use (NSSU) is a nationwide survey commissioned by the Taiwan Food and Drug Administration (TFDA), the Ministry of Health and Welfare in Taiwan. More details of the survey were available elsewhere for the 2014 survey [37] and 2018 survey [39]. Briefly, the target population of each survey was noninstitutionalized residents, 12–64 years old, from 20 counties and cities in Taiwan. To ensure representativeness, participants were selected through a stratified, multistage, probability proportional to size (PPS) random sampling method. During the household interviews, field workers provided participants with an overview of the study, obtained written informed consent, and guided them in using a tablet computer for the computer-assisted self-interview (CASI) process. The survey enrolled 17,837 participants in 2014 (with a response rate of 62.2%) and 18,626 participants in 2018 (with a response rate of 64.6%). More detailed information, including the background, sampling method, and study design, has been reported elsewhere for the 2014 [37] and 2018 [39] surveys. The distributions of sociodemographic characteristics of the participants in the 2014 and 2018 national surveys were similar to the counterparts of the whole population of the nation, as shown in Table S1 in the supplementary material. The two surveys were approved by the Research Ethics Committee of the National Taiwan University Hospital (approval numbers: 201309034RINB in 2014 and 201802031RINB in 2018). Informed written consent was obtained from all participants and their legal guardian if a participants was adolescent. All methods were performed in accordance with the relevant guidelines and regulations.

### Measurements

Participants anonymously completed a computer-assisted self-interview on tablet computers. The questionnaire used in the survey consisted mainly of questions on the use of psychoactive substances, including tobacco, cigarettes, e-cigarettes, alcohol, areca nuts, prescription drugs (sedatives-hypnotics, analgesics, and stimulants), and illicit drugs. Information regarding sociodemographic characteristics and depression symptoms, among others, was also collected.

#### Definitions of use and NMU of sedatives-hypnotics

Participants were asked about the lifetime use of several categories of sedatives-hypnotics, including 10 types of BZDs (triazolam, alprazolam, fludiazepam, diazepam, flunitrazepam, estazolam, brotizolam, midazolam, clonazepam, and lorazepam) and 3 types of Z-drugs (zolpidem, zopiclone, and zaleplon). For those reporting to ever use sedatives-hypnotics, further questions about the last time use, use of dosage and frequency, and the sources of medication and motives of use would be asked. If the last use of sedatives-hypnotics was within the past year, a respondent’s past year use was coded as presence. For those with past-year use of sedatives-hypnotics, NMU was defined as using sedatives-hypnotics without a doctor’s prescription as well as using sedatives-hypnotics more frequently or at a higher dose than prescribed.

#### Sociodemographic characteristics

Several sociodemographic characteristics were evaluated in the questionnaire, including sex, age, marital status, education level, and residence for all participants. For this study, age was stratified into three strata, including adolescents (12–17 years old), young adults (18–39 years old), and middle-aged adults (40 to 64 years old). Marital status was classified into married, divorced/widowed, and single. Educational level was divided into college or above, senior high school, and junior high school or below. The urbanicity of residence was classified into urban, suburban, and rural. The occupation was divided into four groups: group I (other occupations), group II (service and sales workers), group III (plant and machine operators and assemblers, elementary laborers), and group IV (unemployed or retired, including adolescents).

#### Problematic substance use

In addition, the degree of nicotine dependence was assessed using the 6-item Fagerstrom Test for Nicotine Dependence (FTND) [40], with a cutoff score of 4 validated in male Taiwanese smokers [41]. Alcohol use problems were examined using the Alcohol Use Disorders Identification Test (AUDIT) [42], in which three strata (i.e., 0–7, 8–13, and 14 or more) were derived from a stratum-specific likelihood ratio test among inpatients of a general hospital in Taipei [43]. Problematic drug use was measured using the 20-item Drug Abuse Screening Test (DAST) [44], and its validity has been demonstrated in psychiatric outpatients [45, 46].

#### Depression

Depression was assessed using the Chinese version 20-item of the Center for Epidemiologic Studies Depression Scale (CES-D) [47]. CES-D scores of 0–28 were considered low depression scores, and scores of 29–60 were considered medium/high depression scores [48].

### Statistical analyses

To account for the complex sampling design, the prevalence data were estimated using the PROC SURVEYFREQ of SAS 9.4 (SAS Institute Inc., Cary, NC, USA). To compare the prevalence between the two waves, we pooled the two waves of the survey into a dataset and used PROC SURVEYLOGISTIC to conduct multivariable logistic regression analysis, and the difference-in-differences (DID) in prevalence was examined with an interaction term between survey year and sex. Statistical significance was set at P value < 0.05.

## Results

### Changes in past-year use and NMU of sedatives-hypnotics

For the whole sample, the weighted prevalence rates of past-year use of sedatives-hypnotics were 5.46% in 2014 and 5.23% in 2018, and those of NMU were 0.71% in 2014 and 0.67% in 2018, with neither showing significant changes over time (Table 1). However, when the samples were divided into adolescents and adults, only adolescents had significant increases in both use, from 0.42% (SE: 0.16%) to 0.80% (SE: 0.20%), and NMU, from 0.16% (SE: 0.14%) to 0.39% (SE: 0.15%), of sedatives-hypnotics. Owing to the small number of adolescents reporting such use, we could not examine changes in sex-specific strata or other sociodemographic subgroups over time.

**Table 1:** Comparison of the prevalence of past-year use and nonmedical use (NMU) of sedatives-hypnotics between the 2014 and 2018 waves of the national survey in Taiwan

	Sample size			Past-year use of sedatives-hypnotics								Past-year NMU of sedatives-hypnotics						
	2014	2018		2014			2018		Changes (%)	DID (%)		2014			2018		Changes (%)	DID (%)
Variable	N	N		n	%_wt_ (SE)	n		%_wt_ (SE)				n	%_wt_ (SE)	n 119 (0.67) 10 (0.28)		%_wt_ (SE)		
Total	17,837	18,626		836	5.46 (0.25)	896		5.23 (0.22)	-0.23	-		100	0.71 (0.10)	119		0.67 (0.08)	-0.04	*-*
*Stratification by adulthood*																		
Adolescents	4445	3598		15	0.42 (0.16)	22		0.80 (0.20)	0.38*	-		2	0.16 (0.14)	10		0.39 (0.15)	0.25*	*-*
Adults	13,392	15,028		821	6.00 (0.27)	874		5.63 (0.24)	-0.37	-		98	0.77 (0.11)	109		0.70 (0.09)	-0.07	*-*
*Stratification by age groups × sex*																		
18–39 years																		
Male	2921	3366		80	3.07 (0.47)	71		2.29 (0.33)	-0.78¶	Ref		18	0.84 (0.28)	7		0.18 (0.08)	-0.66*	Ref
Female	2815	3144		94	2.91 (0.36)	114		3.81 (0.47)	0.90¶	1.68*		24	0.72 (0.17)	24		0.62 (0.18)	-0.*10*	0.57
40–64 years																		
Male	3695	4115		270	7.48 (0.60)	284		6.56 (0.49)	-0.92	Ref		24	0.81 (0.22)	30		0.74 (0.18)	-0.07	Ref
Female	3961	4403		377	9.99 (0.66)	405		9.02 (0.55)	-0.97	-0.05		32	0.73 (0.17)	48		1.14 (0.20)	0.41¶	0.47

Note: N = unweighted overall number; n = unweighted number; %_wt_ = weighted prevalence; SE = standard error; DID = difference-in-differences of prevalence

*P < 0.05 for a z-test comparing two prevalence rates or a Wald chi-square test for the DID; ¶ borderline significant at 0.05 < *P* < 0.06

We then stratified the adults into young and middle-aged individuals and further stratified them into males and females. For past-year use of sedatives-hypnotics, young adult males and young adult females had a significant difference-in-differences from 2014 to 2018, with a decrease for males (from 3.07 to 2.29%) and an increase for females (from 2.91 to 3.81%). Meanwhile, for the past-year NMU of sedatives-hypnotic, neither young adults nor middle-aged adults had a significant DID between males and females from 2014 to 2018. Nevertheless, two age groups showed significant changes in one sex in the prevalence of NMU, with a decrease for males aged 18–39 years and an increase for females aged 40–64 years.

### Sociodemographic subgroup analysis of past-year use in young adults

Before we conducted subgroup analyses, we examined the correlates in sociodemographic characteristics with past-year use of sedatives-hypnotics in each survey, first for any sedatives-hypnotics (Table S2.a) and then stratified into BZDs (Table S2.b) and Z-drugs (Table S2.c in the supplementary material), and the correlates in other substances use and depression with past-year use of sedatives-hypnotics in each survey, first for any sedatives-hypnotics (Table S3.a) and then stratified into BZDs (Table S3.b) and Z-drugs (Table S3.c in the supplementary material). In general, those sociodemographic correlates were similar in the 2014 and 2018 surveys.

We then explored which sociodemographic subgroups within young adult males and females had significant changes from 2014 to 2018 in the past-year use of sedatives-hypnotics (Table 2). For young adult males, a decrease in the prevalence of past-year use occurred in those living in urban areas (-2.41%), tobacco users (-1.89%), and areca nut users (-2.87%). For young adult females, the increase in the prevalence of past-year use occurred in those who were single (1.09%), had an educational level of junior high school or below (6.59%), were unemployed (2.79%), lived in urban areas (2.88%), tobacco users (7.10%), and alcohol users (1.84%).

**Table 2:** Changes in the prevalence of past-year use of sedatives-hypnotics from 2014 to 2018 in subgroups stratified by sociodemographic data and substance use among young adults (aged 18–39 years), stratified by sex

	Past-year use of sedatives-hypnotics in young adult males									Past-year use of sedatives-hypnotics in young adult females							
	2014			2018						2014			2018				
Variables	N	n	%_wt_		N	n	%_wt_	Change		N	n	%_wt_		N	n	%_wt_	Change
Marital status																	
Married	718	16	2.40	797		13	1.29	-1.11		11,299	39	3.06	1141		39	3.68	0.62
Divorced or widowed	80	9	15.06	77		8	13.24	-1.82		88	11	10.97	90		12	11.75	0.78
Single	2123	55	2.93	2492		50	2.31	-0.62		1598	44	2.39	1913		63	3.48	1.09¶
Education																	
>College	1760	44	1.93	2097		41	1.75	-0.18		1846	60	2.87	2161		70	3.27	0.40
Senior high	958	22	3.55	1083		22	2.36	-1.19		775	28	3.20	822		34	4.20	1.00
<Junior high	203	14	10.32	186		8	8.23	-2.09		194	6	2.10	161		10	8.69	6.59*
Occupation^a^																	
Group I	1412	34	2.27	1602		29	1.51	-0.76		1032	44	3.78	1180		42	3.04	-0.74
Group II	537	14	3.33	524		13	3.13	-0.20		760	25	3.24	904		35	4.38	1.14
Group III	279	9	5.45	411		8	2.87	-2.58		104	4	3.75	128		6	4.24	0.49
Group IV	693	23	3.48	829		21	3.04	-0.44		919	21	1.42	932		31	4.21	2.79*
Urbanicity																	
Urban	364	11	4.23	535		11	1.82	-2.41*		388	11	1.95	472		18	4.83	2.88*
Suburban	2094	59	2.79	2266		49	2.46	-0.33		2049	69	3.10	2158		73	3.08	-0.02
Rural	463	10	2.12	565		11	2.02	-0.10		378	14	4.27	514		23	5.95	1.68
Tobacco use	1003	43	5.44	943		28	3.55	-1.89*		196	22	8.85	202		33	15.95	7.10*
Alcohol use	1846	54	3.44	1932		47	2.59	-0.85		1329	66	3.97	1438		83	5.81	1.84*
Areca nut use	409	22	6.26	427		12	3.39	-2.87¶		21	1	2.69	40		4	4.35	1.66
Prescription analgesic use	175	13	9.23	109		11	9.65	0.42		205	16	5.72	119		14	11.34	5.62

^a^Group I = other occupations; II = service and sales workers; III = plant and machine operators and assemblers, elementary laborers; and IV = unemployed or retired

*P < 0.05 for a z-test comparing two proportions for changes (%), and ¶ at 0.05 < *P* < 0.06

### Sociodemographic subgroup analysis of past-year NMU

For the two age-sex strata with significant changes in the prevalence of NMU of sedatives-hypnotics over time, we also examined the corresponding changes within sociodemographic subgroups (Table 3). For young adult males, their decreases in the NMU of sedatives-hypnotics occurred pervasively in many sociodemographic subgroups as well as users of tobacco, alcohol, and areca nut. For middle-aged females, their increase in the NMU of sedatives-hypnotics occurred mainly for those being married, having an occupation as professional, and users of alcohol.

**Table 3:** Changes in the prevalence of past-year nonmedical use (NMU) of sedatives-hypnotics from 2014 to 2018 in subgroups stratified by sociodemographic data and substance use among males aged 18–39 years and females aged 40–64 years

	Past-year NMU of sedatives-hypnotics in young adult males									Past-year NMU of sedatives-hypnotics in middle-aged females							
	2014			2018						2014			2018				
Variables	N	n	%_wt_		N	n	%_wt_	Change		N	n	%_wt_		N	n	%_wt_	Change
Marital status																	
Married	718	2	0.18	797		0	0.00	-0.18		3042	20	0.64	3379		37	1.18	0.54*
Divorced or widowed	80	5	8.49	77		1	0.58	-7.91*		600	9	0.93	622		8	1.34	0.41
Single	2123	11	0.83	2492		6	0.24	-0.59*		319	3	1.19	402		3	0.50	-0.69
Education																	
> College	1760	6	0.38	2097		5	0.15	-0.23		996	6	0.40	1327		13	1.06	0.66
Senior high	958	8	1.44	1083		2	0.28	-1.16*		1371	11	0.78	1637		16	1.03	0.25
< Junior high	203	4	2.20	186		0	0.00	-2.20*		1594	15	0.95	1439		19	1.35	0.40
Occupation^a^																	
Group I	1412	5	0.52	1602		4	0.18	-0.34		1101	7	0.39	1340		20	1.47	1.08*
Group II	537	4	0.62	524		3	0.58	-0.04		887	9	1.09	920		10	1.34	0.25
Group III	279	3	2.97	411		0	0.00	-2.97*		294	4	1.23	277		0	0.00	-1.23
Group IV	693	6	0.66	829		0	0.00	-0.66*		1679	12	0.69	1866		18	0.97	0.28
Urbanicity																	
Urban	364	4	1.46	535		1	0.24	-1.22*		602	7	0.75	678		7	1.46	0.71
Suburban	2094	12	0.63	2266		6	0.21	-0.42*		2792	19	0.71	2999		32	1.13	0.42
Rural	463	2	0.05	565		0	0.00	-0.05		567	6	0.83	726		9	0.84	0.01
Tobacco use	1003	10	1.38	943		5	0.49	-0.89*		169	8	5.56	198		4	1.70	-3.86*
Alcohol use	1846	12	1.11	1932		5	0.25	-0.86*		1090	11	0.86	1178		23	2.37	1.51*
Areca nut use	409	7	1.83	427		0	0.00	-1.83*		72	0	0.00	106		2	1.76	1.76
Prescription analgesic use	175	4	2.45	109		0	0.00	-2.45		269	5	1.14	210		4	2.10	0.96

^a^Group I = other occupations; II = service and sales workers; III = plant and machine operators and assemblers, elementary laborers; and IV = unemployed or retired

*P < 0.05 for a z-test comparing two proportions for changes (%), and ¶ at 0.05 < *P* < 0.06

### Trends in the use and NMU by different types of sedatives-hypnotics

We then examined past-year use of individual types of sedatives-hypnotics from 2014 to 2018 for those who remembered the name of sedatives-hypnotics they had used (Table 4). From 2014 to 2018, BZDs as a whole exhibited an increase of 24.79% in past-year use prevalence and 29.17% in NMU prevalence. Among the individual BZDs, alprazolam had the most prominent increase, followed by triazolam.

**Table 4:** Changes in the proportions of individual types of sedatives-hypnotics for past-year use and NMU between the 2014 and 2018 waves of the national survey among people who could remember the names of the sedatives-hypnotics that they used in the past year

	Past-year use							Past-year NMU					
	2014 (*N* = 354)			2018 (*N* = 567)		Changes (%)		2014 (*N* = 43)			2018 (*N* = 73)		Changes (%)
	n	%_wt_		n	%_wt_			n	%_wt_		n	%_wt_	
BZDs													
*Total*	*154*	*40.94*		*363*	*65.73*	24.79*		*19*	*30.80*		*45*	*59.97*	29.17*
Triazolam	5	1.24		29	6.02	4.78*		1	1.03		4	5.50	4.47
Alprazolam	50	10.21		144	26.19	15.99*		3	1.92		20	32.07	30.15*
Estazolam	63	16.48		104	19.30	2.82		13	19.57		12	17.30	-2.27
Brotizolam	19	5.90		24	4.06	-1.85		3	4.35		4	4.87	0.52
Lorazepam	25	6.86		35	5.44	-1.42		3	8.01		2	0.86	-7.15
Z-drugs													
*Total*	*241*	*70.90*		*205*	*35.00*	-35.90*		*30*	*70.12*		*28*	*38.67*	-31.45*
Zolpidem	229	68.78		166	28.46	-40.32*		29	69.26		24	31.16	-38.10*
Zopiclone	9	2.62		45	7.14	4.53*		0	0.00		5	7.94	7.94*
Zaleplon	15	3.93		8	1.24	-2.68*		5	6.57		0	0.00	-6.57*

Note: N = unweighted overall number; n = unweighted number; %_wt_=weighted prevalence

*P < 0.05 for a z-test comparing two proportions for changes (%)

In contrast, Z-drugs as a whole exhibited a decrease of 35.90% in past-year use prevalence and 31.45% in NMU prevalence. Among the individual Z-drugs, zolpidem had the most prominent decrease, followed by zaleplon.

### Sources of and motives for NMU of sedatives-hypnotics

Table 5 shows the sources of and motives for past-year NMU of sedatives-hypnotics in the two waves of the national survey. From 2014 to 2018, the source from pharmacy showed a significant decline of 35.10%, and the source from doctor prescriptions also exhibited a significant decrease of 13.31%. In contrast, the source from relatives or friends exhibited a significant rise of 59.52%. For the motives, no significant change was observed from 2014 to 2018. The most common motive for NMU of sedatives-hypnotics was self-medication in both waves of the national survey, with rates of 98.11% in 2014 and 96.41% in 2018.

**Table 5:** Sources of and motives for nonmedical use (NMU) of sedatives-hypnotics in the 2014 and 2018 waves of the national survey in Taiwan

		NMU of sedatives-hypnotics					
		2014			2018		Changes (%)
		n	%_wt_		n	%_wt_	
*Total*		*100*	*0.71*		*119*	*0.67*	*-0.04*
Sources							
	Pharmacy	52	53.54		27	18.44	-35.10*
	Places of entertainment	3	2.02		0	0.00	-2.02
	Relatives or friends	0	0.00		61	59.52	59.52*
	Doctors’ prescriptions	36	35.86		32	22.55	-13.31*
Motives							
	Self-medication	97	98.11		115	96.41	-1.70
	Recreational use or others	8	5.32		5	2.23	-3.09

Note: n = unweighted number; %_wt_=weighted prevalence

Totals may exceed 100% because response categories were “select all that apply”.

*P < 0.05 for a z-test comparing two proportions for changes (%)

## Discussion

By comparing the results from the 2014 with the 2018 national surveys among Taiwanese individuals aged 12 to 64 years, we examined the trends for past-year use and NMU of sedatives-hypnotics over time at the individual level, representing the first study in Asia to conduct such a comparison. Among the age-sex strata examined, young adult males had decreasing trends for both past-year use and NMU of sedatives-hypnotics. In contrast, adolescents and young adult females had an increasing trend for past-year use, whereas adolescents and middle-aged adult females showed an increasing trend for past-year NMU of sedatives-hypnotics. Among the strata of young and middle-aged adult females, the increasing trends for past-year use and NMU, respectively, were found to occur mainly in certain sociodemographic subgroups, with alcohol users being the overlapping subgroup. When individual types of sedative-hypnotics were examined, BZDs as a whole had an increasing trend, whereas Z-drugs as a whole showed decreasing trends for the proportions of both past-year use and NMU. In addition, an increased proportion of NMU sourcing from relatives or friends was evident. Meanwhile, self-medication remained the most common motive for NMU of sedatives-hypnotics. Our findings have implications for the regulation of sedative-hypnotic use and the prevention of NMU.

Our finding of a decreasing trend of the past-year use of sedatives-hypnotics in young adult males was similar to those found in young adult males in European countries, e.g., people aged 18–25 years old in Finland from 2006 to 2014 [49], those aged 20–25 years old in Sweden, Norway, and Denmark from 2012 to 2018 [50], and those aged 20 to 29 years old in Norway from 2004 to 2019 [51]. This decreasing trend for past-year use of sedatives-hypnotics over time in Taiwanese young adult males may have led to their decreasing trend for past-year NMU, indicating a fair response to the stricter regulations adopted by the government since 2012 and 2013 [35, 36].

Against this backdrop, the reasons why adolescents, young adult females, and middle-aged adult females failed to respond to these tightened regulations warrant thorough discussion. First, our finding of an increasing trend in the past-year use of sedatives-hypnotics among adolescents is particularly of concern, as alerted in a recent US study that many prescriptions of BZDs lacked evidence of pediatric efficacy [52]. Although the Taiwanese Food and Drug Administration approved benzodiazepines for the treatment of selected psychiatric conditions in adults, benzodiazepines remain unapproved for pediatric use outside of epilepsy and seizures [53–55]. The prevalence of past-year use of sedatives-hypnotics in Taiwanese adolescents in 2014 (0.42%) was close to the lower end of annual prescription prevalence, whereas that in 2018 (0.80%) was close to or higher than the upper end of such estimates in North America (0.3-0.5%) [56], Europe (0.2-0.9%) [57], and Sweden (0.43-0.46%) [9]. An increasing trend for past-year use of sedatives-hypnotics over time in Taiwanese adolescents is also found in the annual prescription prevalence among adolescents in Sweden (from 2006 to 2013) [9] and Canada (from 1996 to 2012) [56]. Nevertheless, cross-European comparisons among adolescents from 2001 to 2009 revealed a mixed picture, with Spain and the UK showing increases, whereas the Netherlands, Germany, and Denmark showed decreases [58], and a recent study based on dispensed prescriptions from 2004 to 2019 in Norway found that benzodiazepines use remained relatively stable across the period (< 0.8%) [51].

Second, Taiwanese adolescents’ NMU of sedatives-hypnotics showed an increasing trend over time. Of note, the proportion of NMU out of past-year use of sedatives-hypnotics in adolescents (0.16/0.42 = 38.1% in 2014 and 0.39/0.80 = 48.8% in 2018) was much higher than that in adults (12.8% in 2014 and 12.4% in 2018). This vulnerability to NMU in adolescents reflects an important observation in the past two decades that adolescents worldwide have become an age group at high risk for NMU of sedatives-hypnotics [59–61]. The prevalence estimates of the past-year NMU of sedatives-hypnotics among Taiwanese adolescents (0.16% in 2014 and 0.39% in 2018) were still lower than those in other industrial countries, e.g., the past-year NMU being 1.7-3.0% for sedatives and 0.4-2.0% for tranquilizers in the U.S. between 2001 and 2011 [62] as well as 1.2% for sedatives in five European countries in 2014 [63]. However, industrial countries’ trends over time in adolescents’ NMU of sedatives-hypnotics differed, e.g., those for US adolescents since 2016 were stable [64, 65], and those for Spanish adolescents from 2004 to 2014 showed an increasing trend [66].

Third, young adult females and middle-aged adult females in Taiwan had increasing trends over time for the past-year use and NMU, respectively, of sedatives-hypnotics. Some clues for such increases might be obtained from our subgroup analyses. That is, the subgroups with an increasing trend in the past-year use of sedatives-hypnotics seem to implicate those young adult females with fewer resources, such as being single, having a low educational level, being unemployed, and having substance use (tobacco and alcohol). For comparison, the increase in the past-year NMU of sedatives-hypnotics in the middle-aged adult females was mainly among those who were being married, having an occupation as professionals, and users of alcohol, implicating the middle-aged adult females of these subgroups having more social establishment than those of the subgroups of young adult females. One plausible explanation is due to multiple roles imposed on those middle-aged adult females, i.e., being simultaneously a wife, having an occupation as professionals, and having work-related alcohol use such that they developed a pattern of NMU of sedatives-hypnotics to counter the sleep problem probably being worsened as a result of the combination of these conflicting roles. The identification of these sociodemographic subgroups with an increasing trend of past-year use or NMU can help focus the target for age-sex strata-based prevention strategies.

Regarding the use of different types of sedatives-hypnotics reported by respondents from 2014 to 2018, our finding of an increasing trend of BZDs but a decreasing trend of Z-drugs is likely to result from the Taiwanese government’s new regulations about their prescriptions [35] and request for explicit warnings [36] in response to the rising concerns over the serious adverse effects of Z-drugs, e.g., sleep driving and sleep conversations [67]. Such findings have demonstrated the effectiveness of the restrictions of Z-drug prescriptions, warranting future investigations to determine whether these policies continue to help decrease the prescription of Z-drugs.

Another finding about the shift of the source of NMU of sedatives-hypnotics in Taiwan from pharmacies and physicians’ prescriptions to that from relatives or friends is compatible with the findings from recent systematic reviews [27, 29], which dubbed such a source “social supply.” In contrast, self-medication remained the most common motive for NMU of sedatives-hypnotics in both surveys (98.11% in 2014 and 96.41% in 2018) in Taiwan, similar to the finding of a previous review [29]. Since Asian adults with mental health problems (e.g., depression and anxiety) have been found to prefer seeking help from family or friends rather than from mental health professionals [68], a culture-tailored approach may be needed to decrease the NMU of sedatives-hypnotics in Asian countries.

Our findings have implications for improving the regulations of sedative-hypnotic use in Taiwan. To mitigate the increasing trend over time for past-year use or NMU of sedatives-hypnotics, new preventive measures should target specific age-sex strata rather than relying on a generic approach. For adolescents, an important reason for their vulnerability to use or NMU of sedatives-hypnotics is their lack of relevant knowledge, or so-called medication literacy. A 2016 survey in Taiwan found that nearly half of school-attending adolescents reported self-medication for a variety of illnesses in the past year, with 10–30% of these adolescents having inappropriate self-medication behaviors, such as not receiving advice from a health provider, using excessive dosages, and not reading drug labels or instructions [69]. Thus, developing an education program on medication literacy directed at both adolescents and their families is critical to curtail the increasing trends of sedative-hypnotic use/NMU in this part of the population [70]. In addition, when treating adolescents, clinicians might opt for alternatives with lower misuse potential, such as selective serotonin reuptake inhibitors for anxiety and melatonin for insomnia [51]. For the young adult female population, where the results of subgroup analyses pointed to those with fewer resources, those with insomnia might not be able to afford to undergo nonpharmacological intervention, such as cognitive‒behavioral therapy, although this form of therapy has been recommended in many guidelines as the first choice for chronic insomnia [1]. Hence, decreasing sedative-hypnotic use in young adult females will require improving the availability of cognitive‒behavioral therapy. Furthermore, a common sociodemographic subgroup in which both young adult females (for past-year use) and middle-aged adult females (for NMU) had an increasing trend for sedative-hypnotic use over time is the subgroup of females with alcohol use, which echoes a recent finding that from 2014 to 2018, Taiwanese females had an increasing trend of harmful alcohol use, particularly those aged 18 to 29 years old [71]. One explanation is the concurrent use of sedatives-hypnotics and alcohol [72] because both sedatives-hypnotics and alcohol have sedative effects and can help induce a rapid onset of sleep [73]. Hence, a common preventive approach for decreasing the use of alcohol and sedatives-hypnotics is important, such as a policy of comprehensive restriction on alcohol advertising, promotion and sponsorship [71].

This study had limitations. First, this study did not include individuals aged 65 years or older, the age group with the highest prevalence of sedative-hypnotic use. Hence, our whole-sample estimates did not represent those of the general population. Second, only two waves of the survey, conducted 4 years apart, were compared, which might be too short to capture changes in certain age-sex strata. Nevertheless, our findings in this study provide essential insights up to 2018, and once the data of 2023 National Survey of Substance Use become publicly available, these insights will be invaluable for assessing the effectiveness of recent domestic regulations. Third, this study lacks detailed information about mental conditions such as insomnia or anxiety in the participants. Hence, we did not know the real motive for their use of sedatives-hypnotics. Last, most of our participants had difficulty identifying the type of sedatives-hypnotics that they had previously used; thus, the trends for different drug types were restricted to those who could identify drug names.

## Conclusions

From the 2014 to 2018 national surveys in Taiwan, the prevalence of past-year use or NMU of sedatives-hypnotics exhibited differential trends over time in different age-sex strata of the population under the context of tightened government regulations on prescribing sedatives-hypnotics since 2012. Young adult males showed decreasing trends for both past-year use and NMU of sedatives-hypnotics, whereas adolescents and young adult females showed an increasing trend in past-year use, and adolescents and middle-aged adult females had increasing trends for past-year NMU of sedatives-hypnotics. Increasing medication literacy among adolescents and improving regulations targeting specific age-sex strata are indicated to curtail these increasing trends over time for the use or NUM of sedatives-hypnotics.

## Electronic supplementary material

Below is the link to the electronic supplementary material.

Supplementary Material 1

## Data Availability

The datasets analyzed for the current study are not publicly available due to the requirement of obtaining official permission to access the data but are available from the corresponding author upon reasonable request.
