# Supplementary material for: Trends in the use and nonmedical use of sedatives-hypnotics in the population aged 12 to 64 years in Taiwan: a comparative analysis of the national surveys in 2014 and 2018
Source: BMC Public Health. 2024 Nov 25;24:3262. doi: 10.1186/s12889-024-20778-1 (PMC11587671; doi:10.1186/s12889-024-20778-1)
Supplement: Supplementary file 1 — Supplementary Material 1 [file 12889_2024_20778_MOESM1_ESM.docx]

**Supplementary Information**

Table S1. Sociodemographic characteristics of participants in the 2014 and 2018 National Survey of Substance Use, respectively, in Taiwan.

Table S2.a Correlates in sociodemographic characteristics with past-year use of any sedatives-hypnotics among participants in the 2014 and 2018 waves of the national survey.

Table S2.b Correlates in sociodemographic characteristics with past-year use of benzodiazepines (BZDs) among participants in the 2014 and 2018 waves of the national survey.

Table S2.c Correlates in sociodemographic characteristics with past-year use of Z-drugs among participants in the 2014 and 2018 waves of the national survey.

Table S3.a Correlates in other substances use and depression with past-year use of sedatives-hypnotics among participants in the 2014 and 2018 waves of the national survey.

Table S3.b Correlates in other substances use and depression with past-year use of benzodiazepines (BZDs) among participants in the 2014 and 2018 waves of the national survey.

Table S3.c Correlates in other substances use and depression with past-year use of Z-drugs among participants in the 2014 and 2018 waves of the national survey.

Table S1. Sociodemographic characteristics of participants in the 2014 and 2018 National Survey of Substance Use, respectively, in Taiwan.

|  | 2014 National Survey | |  | Taiwan population in 2014^a^ % |  | 2018 National Survey | |  | Taiwan population in 2018^a^ % |
| --- | --- | --- | --- | --- | --- | --- | --- | --- | --- |
| Variable | N | Weighted % (SE) |  |  |  | N | Weighted % (SE) |  |  |
| Total | 17837 |  |  |  |  | 18626 |  |  |  |
| Gender |  |  |  |  |  |  |  |  |  |
| Male | 8922 | 50.12 (0.54) |  | 50.15 |  | 9380 | 50.10 (0.49) |  | 50.10 |
| Female | 8915 | 49.88 (0.54) |  | 49.85 |  | 9246 | 49.90 (0.49) |  | 49.90 |
| Age group |  |  |  |  |  |  |  |  |  |
| 12-17 | 4445 | 9.77 (0.22) |  | 9.26 |  | 3598 | 8.21 (0.18) |  | 8.21 |
| 18-24 | 1830 | 11.79 (0.35) |  | 12.44 |  | 2100 | 12.06 (0.32) |  | 12.08 |
| 25-29 | 1115 | 9.66 (0.38) |  | 8.77 |  | 1373 | 9.04 (0.30) |  | 9.02 |
| 30-34 | 1374 | 11.14 (0.36) |  | 10.82 |  | 1428 | 9.46 (0.31) |  | 9.48 |
| 35-39 | 1417 | 10.50 (0.35) |  | 10.93 |  | 1609 | 11.46 (0.35) |  | 11.43 |
| 40-44 | 1500 | 10.00 (0.33) |  | 9.88 |  | 1654 | 10.43 (0.32) |  | 10.44 |
| 45-49 | 1607 | 10.23 (0.34) |  | 10.16 |  | 1618 | 10.18 (0.30) |  | 10.17 |
| 50-54 | 1700 | 10.23 (0.32) |  | 10.31 |  | 1735 | 10.28 (0.30) |  | 10.30 |
| 55-59 | 1479 | 9.20 (0.31) |  | 9.44 |  | 1891 | 10.09 (0.28) |  | 10.03 |
| 60-64 | 1370 | 7.48 (0.27) |  | 7.97 |  | 1620 | 8.80 (0.27) |  | 8.85 |
| N = unweighted number; SE = standard error.  ^a^Source: Department of Statistics, Ministry of the Interior | | | | | | | | | |

Table S2.a Correlates in sociodemographic characteristics with past-year use of any sedatives-hypnotics among participants in the 2014 and 2018 waves of the national survey.

|  | Past-year use of any sedatives-hypnotics, 2014 | | | |  | Past-year use of any sedatives-hypnotics, 2018 | | | |
| --- | --- | --- | --- | --- | --- | --- | --- | --- | --- |
| Variable | N | n (%_wt_) | OR (95% CI) | aOR (95% CI)^a^ |  | N | n (%_wt_) | OR (95% CI) | aOR (95% CI)^a^ |
| Total | 1783777 | 836 (5.46) | - | - |  | 18626 | 896 (5.23) | - | - |
|  |  |  |  |  |  |  |  |  |  |
| Gender |  |  |  |  |  |  |  |  |  |
| Male | 8922 | 356 (4.83) | Ref | Ref |  | 9380 | 364 (4.24) | Ref | Ref |
| Female | 8915 | 480 (6.08) | 1.28 (1.05-1.55)* | 1.07 (0.87-1.32) |  | 9246 | 532 (6.22) | 1.50 (1.26-1.79)*** | 1.30 (1.07-1.57)** |
| Age group (yrs) |  |  |  |  |  |  |  |  |  |
| 12-17 | 4445 | 15 (0.42) | Ref | Ref |  | 3598 | 22 (0.80) | Ref | Ref |
| 18-39 | 5736 | 174 (2.99) | 7.28 (3.33-15.90)*** | 13.22 (5.85-29.87)*** |  | 6510 | 185 (3.04) | 3.90 (2.27-6.70)*** | 7.92 (4.36-14.39)*** |
| 40-64 | 7656 | 647 (8.75) | 22.65 (10.57-48.56)*** | 32.43 (14.17-74.23)*** |  | 8518 | 689 (7.81) | 10.56 (6.31-17.69)*** | 18.68 (10.21-34.19)*** |
| Marital status |  |  |  |  |  |  |  |  |  |
| Married | 7856 | 526 (6.54) | Ref | Ref |  | 8479 | 532 (5.95) | Ref | Ref |
| Divorced/widowed | 1103 | 136 (13.33) | 2.20 (1.68-2.88)*** | 1.87 (1.41-2.47)*** |  | 1216 | 154 (11.55) | 2.06 (1.61-2.64)*** | 1.77 (1.37-2.28)*** |
| Single | 8878 | 174 (2.88) | 0.42 (0.33-0.54)*** | 1.05 (0.77-1.43) |  | 8931 | 210 (3.26) | 0.53 (0.43-0.66)*** | 1.15 (0.86-1.52) |
| Educational level |  |  |  |  |  |  |  |  |  |
| $\geq$ College | 5966 | 258 (3.77) | Ref | Ref |  | 7026 | 291 (3.98) | Ref | Ref |
| Senior high | 6593 | 287 (5.92) | 1.61 (1.28-2.03)*** | 1.48 (1.15-1.92)** |  | 5184 | 309 (5.84) | 1.50 (1.21-1.85)*** | 1.17 (0.94-1.45) |
| $\leq$ Junior high | 5278 | 291 (7.94) | 2.20 (1.75-2.77)*** | 1.62 (1.21-2.17)** |  | 6416 | 296 (6.69) | 1.73 (1.39-2.15)*** | 1.45 (1.11-1.89)** |
| Employment |  |  |  |  |  |  |  |  |  |
| No | 8496 | 373 (6.51) | Ref | Ref |  | 8065 | 400 (6.86) | Ref | Ref |
| Yes | 9341 | 463 (4.87) | 0.73 (0.61-0.89)** | 0.61 (0.50-0.75)*** |  | 10561 | 496 (4.38) | 0.62 (0.52-0.74)*** | 0.56 (0.46-0.68)*** |
| Urbanicity |  |  |  |  |  |  |  |  |  |
| Urban | 2290 | 132 (6.27) | Ref | Ref |  | 2674 | 125 (5.27) | Ref | Ref |
| Suburban | 12739 | 582 (5.16) | 0.81 (0.64-1.04) | 0.82 (0.63-1.06) |  | 12638 | 609 (5.17) | 0.98 (0.75-1.27) | 0.97 (0.74-1.26) |
| Rural | 2808 | 122 (5.34) | 0.84 (0.62-1.14) | 0.76 (0.55-1.06) |  | 3314 | 162 (5.42) | 1.03 (0.75-1.41) | 0.96 (0.70-1.33) |
| ^a^Adjusted odds ratio and its 95% confidence interval controlling for all the sociodemographic variables in this table.  *P<0.05; **P<0.01; ***P<0.001 | | | | | | | | | |

|  | Past-year use of BZDs, 2014 | | | |  | Past-year use of BZDs, 2018 | | | |
| --- | --- | --- | --- | --- | --- | --- | --- | --- | --- |
| Variable | N | n (%_wt_) | OR (95% CI) | aOR (95% CI)^a^ |  | N | n (%_wt_) | OR (95% CI) | aOR (95% CI)^a^ |
| Total | 17837 | 154 (0.96) | - | - |  | 18626 | 363 (2.13) | - | - |
|  |  |  |  |  |  |  |  |  |  |
| Gender |  |  |  |  |  |  |  |  |  |
| Male | 8922 | 72 (0.91) | Ref | Ref |  | 9380 | 150 (1.69) | Ref | Ref |
| Female | 8915 | 82 (1.02) | 1.11 (0.73-1.71) | 0.96 (0.59-1.57)*** |  | 9246 | 213 (2.57) | 1.53 (1.17-2.00)** | 1.31 (0.99-1.74) |
| Age group (yrs) |  |  |  |  |  |  |  |  |  |
| 12-17 | 4445 | 1 (0.01) | Ref | Ref |  | 3598 | 6 (0.24) | Ref | Ref |
| 18-39 | 5736 | 36 (0.60) | 44.5 (5.93-333.30)** | 79.54 (9.50-666.05)** |  | 6510 | 84 (1.18) | 5.01 (1.93-12.99) | 9.26 (3.33-25.79)* |
| 40-64 | 7656 | 117 (1.50) | 111.9 (15.53-806.24)*** | 206.4 (25.9->999.99)*** |  | 8518 | 273 (3.24) | 14.97 (5.56-35.10)*** | 23.40 (8.43-64.92)*** |
| Marital status |  |  |  |  |  |  |  |  |  |
| Married | 7856 | 96 (1.11) | Ref | Ref |  | 8479 | 205 (2.40) | Ref | Ref |
| Divorced/widowed | 1103 | 21 (1.93) | 1.76 (0.94-3.29)** | 1.54 (0.80-2.95) |  | 1216 | 66 (5.06) | 2.17 (1.51-3.10)*** | 1.89 (1.31-2.74)** |
| Single | 8878 | 37 (0.64) | 0.57 (0.35-0.95)** | 1.40 (0.70-2.80) |  | 8931 | 92 (1.29) | 0.53 (0.39-0.73)*** | 1.15 (0.74-1.77) |
| Educational level |  |  |  |  |  |  |  |  |  |
| College | 5966 | 68 (0.83) | Ref | Ref |  | 7026 | 133 (1.74) | Ref | Ref |
| Senior high | 6593 | 44 (0.82) | 0.99 (0.61-1.62) | 0.94 (0.54-1.62) |  | 5184 | 119 (2.25) | 1.30 (0.95-1.78) | 0.99 (0.71-1.37) |
| Junior high | 5278 | 42 (1.42) | 1.72 (1.03-2.88)* | 1.30 (0.58-2.92) |  | 6416 | 111 (2.67) | 1.55 (1.11-2.17)* | 1.22 (0.81-1.86) |
| Employment |  |  |  |  |  |  |  |  |  |
| No | 8496 | 71 (1.18) | Ref | Ref |  | 8065 | 161 (2.85) | Ref | Ref |
| Yes | 9341 | 83 (0.84) | 0.71 (0.46-1.09) | 0.57 (0.36-0.91) |  | 10561 | 202 (1.75) | 0.61 (0.46-0.79)*** | 0.54 (0.40-0.73)*** |
| Urbanicity |  |  |  |  |  |  |  |  |  |
| Urban | 2290 | 24 (0.94) | Ref | Ref |  | 2674 | 52 (2.45) | Ref | Ref |
| Suburban | 12739 | 105 (0.95) | 1.01 (0.57-1.80) | 1.05 (0.55-1.98) |  | 12638 | 247 (2.00) | 0.81 (0.55-1.20) | 0.82 (0.55-1.21) |
| Rural | 2808 | 25 (1.14) | 1.22 (0.62-2.39) | 1.18 (0.53-2.64) |  | 3314 | 64 (2.35) | 0.96 (0.59-1.55) | 0.93 (0.57-1.52) |
| ^a^Adjusted odds ratio and its 95% confidence interval controlling for all the sociodemographic variables in this table.  *P<0.05; **P<0.01; ***P<0.001 | | | | | | | | | |

Table S2.b Correlates in sociodemographic characteristics with past-year use of benzodiazepines (BZDs) among participants in the 2014 and 2018 waves of the national survey.

Table S2.c Correlates in sociodemographic characteristics with past-year use of Z-drugs among participants in the 2014 and 2018 waves of the national survey.

|  | Past-year use of Z-drugs, 2014 | | | |  | Past-year use of Z-drugs, 2018 | | | |
| --- | --- | --- | --- | --- | --- | --- | --- | --- | --- |
| Variable | N | n (%_wt_) | OR (95% CI) | aOR (95% CI)^a^ |  | N | n (%_wt_) | OR (95% CI) | aOR (95% CI)^a^ |
| Total | 17837 | 241 (1.67) | - | - |  | 18626 | 205 (1.13) | - | - |
|  |  |  |  |  |  |  |  |  |  |
| Gender |  |  |  |  |  |  |  |  |  |
| Male | 8922 | 116 (1.68) | Ref | Ref |  | 9380 | 79 (0.82) | Ref | Ref |
| Female | 8915 | 125 (1.66) | 0.99 (0.70-1.39) | 0.85 (0.59-1.23) |  | 9246 | 126 (1.44) | 1.76 (1.23-2.53)** | 1.58 (1.07-2.33)* |
| Age group (yrs) |  |  |  |  |  |  |  |  |  |
| 12-17 | 4445 | 2 (0.06) | Ref | Ref |  | 3598 | 2 (0.17) | Ref | Ref |
| 18-39 | 5736 | 68 (1.19) | 19.64 (3.57-108.06)* | 26.78 (4.65-154.34) |  | 6510 | 43 (0.62) | 3.57 (0.84-15.19) | 4.18 (0.89-19.73) |
| 40-64 | 7656 | 171 (2.44) | 40.67 (7.52-219.92)*** | 46.55 (8.08-268.19)** |  | 8518 | 160 (1.73) | 10.13 (2.50-41.12)*** | 11.87 (2.48-56.75)*** |
| Marital status |  |  |  |  |  |  |  |  |  |
| Married | 7856 | 141 (1.85) | Ref | Ref |  | 8479 | 122 (1.31) | Ref | Ref |
| Divorced/widowed | 1103 | 42 (4.44) | 2.47 (1.53-3.97)*** | 2.30 (1.43-3.70)*** |  | 1216 | 38 (2.65) | 2.05 (1.27-3.32)*** | 1.91 (1.16-3.14)* |
| Single | 8878 | 58 (1.01) | 0.54 (0.36-0.82)*** | 0.99 (0.61-1.60) |  | 8931 | 45 (0.66) | 0.50 (0.32-0.78)*** | 1.06 (0.57-1.97) |
| Educational level |  |  |  |  |  |  |  |  |  |
| College | 5966 | 98 (1.38) | Ref | Ref |  | 7026 | 84 (1.07) | Ref | Ref |
| Senior high | 6593 | 92 (2.05) | 1.50 (1.03-2.19) | 1.48 (0.97-2.25)* |  | 5184 | 72 (1.18) | 1.11 (0.74-1.67) | 0.79 (0.53-1.16) |
| Junior high | 5278 | 51 (1.67) | 1.21 (0.77- 1.90) | 1.03 (0.60-1.78) |  | 6416 | 49 (1.19) | 1.11 (0.70-1.76) | 0.77 (0.45-1.30) |
| Employment |  |  |  |  |  |  |  |  |  |
| No | 8496 | 94 (1.73) | Ref | Ref |  | 8065 | 84 (1.41) | Ref | Ref |
| Yes | 9341 | 147 (1.64) | 0.95 (0.66-1.35) | 0.70 (0.48-1.03) |  | 10561 | 121 (0.99) | 0.70 (0.48-1.00)* | 0.61 (0.42-0.89)** |
| Urbanicity |  |  |  |  |  |  |  |  |  |
| Urban | 2290 | 46 (2.31) | Ref | Ref |  | 2674 | 28 (0.83) | Ref | Ref |
| Suburban | 12739 | 168 (1.46) | 0.63 (0.42-0.94) | 0.64 (0.42-0.98) |  | 12638 | 140 (1.14) | 1.38 (0.83-2.29) | 1.46 (0.87-2.45) |
| Rural | 2808 | 27 (1.45) | 0.62 (0.36-1.08) | 0.59 (0.33-1.08) |  | 3314 | 37 (1.34) | 1.62 (0.85-3.06 | 1.75 (0.91-3.38) |
| ^a^Adjusted odds ratio and its 95% confidence interval controlling for all the sociodemographic variables in this table.  *P<0.05; **P<0.01; ***P<0.001 | | | | | | | | | |

Table S3.a Correlates in other substances use and depression with past-year use of sedatives-hypnotics among participants in the 2014 and 2018 waves of the national survey.

|  | Past-year use of any sedatives-hypnotics, 2014 | | |  | Past-year use of any sedatives-hypnotics, 2018 | | |
| --- | --- | --- | --- | --- | --- | --- | --- |
| Variable | N | n (%_wt_) | aOR (95% CI)^a^ |  | N | n (%_wt_) | aOR (95% CI)^a^ |
| Total | 17837 | 836 (5.46) | - |  | 18626 | 896 (5.23) | - |
|  |  |  |  |  |  |  |  |
| Tobacco | 3001 | 234 (8.15) | 1.95 (1.48-2.57)*** |  | 2955 | 233 (7.85) | 2.04 (1.59-2.62)*** |
| Alcohol | 7292 | 387 (5.42) | 1.21 (0.98-1.48) |  | 7327 | 415 (6.08) | 1.79 (1.47-2.18)*** |
| Binge drinking | 587 | 42 (7.69) | 1.73 (1.05-2.85)* |  | 543 | 33 (6.30) | 1.56 (1.01-2.41)* |
| Areca nuts | 1141 | 86 (8.14) | 1.61 (1.14-2.29)** |  | 1247 | 84 (6.85) | 1.34 (0.98-1.83) |
| Prescription analgesics | 1129 | 142 (12.64) | 2.52 (1.90-3.34)*** |  | 720 | 115 (15.72) | 3.31(2.47-4.44)*** |
| NMU | 498 | 52 (12.30) | 2.67 (1.75-4.08)*** |  | 213 | 20 (9.13) | 1.78 (0.89-3.56) |
| Illicit drug | 37 | 17 (44.28) | 18.94 (9.00-39.85)*** |  | 41 | 16 (50.87) | 24.78 (11.45-53.63)*** |
| FTND score |  |  |  |  |  |  |  |
| Non-user | 13643 | 523 (4.62) | Ref |  | 14354 | 554 (4.23) | Ref |
| 0-3 | 2144 | 143 (6.92) | 1.84 (1.35-2.52)*** |  | 2695 | 202 (7.45) | 2.39 (1.82-3.14)*** |
| ≥4 | 2050 | 170 (8.45) | 2.09 (1.53-2.86)*** |  | 1577 | 140 (9.28) | 2.86 (2.11-3.88)*** |
| AUDIT score |  |  |  |  |  |  |  |
| Non-user | 8828 | 350 (5.16) | Ref |  | 9937 | 391 (4.36) | Ref |
| 0-7 | 7932 | 406 (5.32) | 1.32 (1.06-1.63)* |  | 7742 | 421 (5.82) | 1.69 (1.38-2.08)*** |
| ≥8 | 1077 | 80 (8.25) | 1.81 (1.24-2.63)** |  | 947 | 84 (7.91) | 2.28 (1.62-3.20)*** |
| CES-D stratum |  |  |  |  |  |  |  |
| Medium/high (29-60) | 781 | 126 (18.27) | 4.83 (3.52-6.61)*** |  | 656 | 110 (17.38) | 5.03 (3.67-6.88)*** |
| ^a^Adjusted odds ratio and its 95% confidence interval controlling for all the sociodemographic variables in Table S2.  *P<0.05; **P<0.01; ***P<0.001 | | | | | | | |

Table S3.b Correlates in other substances use and depression with past-year use of benzodiazepines (BZDs) among participants in the 2014 and 2018 waves of the national survey.

|  | Past-year use of BZDs, 2014 | | |  | Past-year use of BZDs, 2018 | | |
| --- | --- | --- | --- | --- | --- | --- | --- |
| Variable | N | n (%_wt_) | aOR (95% CI)^a^ |  | N | n (%_wt_) | aOR (95% CI)^a^ |
| Total | 17837 | 154 (0.96) | - |  | 18626 | 363 (2.13) | - |
|  |  |  |  |  |  |  |  |
| Tobacco | 3001 | 49 (1.72) | 2.69 (1.53-4.70)*** |  | 2955 | 101 (3.26) | 2.12 (1.48-3.04)*** |
| Alcohol | 7292 | 76 (0.95) | 1.10 (0.70-1.73) |  | 7327 | 190 (2.73) | 2.21 (1.64-2.98)*** |
| Binge drinking | 587 | 14 (1.94) | 2.44 (1.04-5.76)* |  | 543 | 16 (2.60) | 1.58 (0.85-2.91) |
| Areca nuts | 1141 | 18 (1.38) | 1.45 (0.74-2.83) |  | 1247 | 32 (2.60) | 1.25 (0.78-1.98) |
| Prescription analgesics | 1129 | 28 (2.80) | 3.12 (1.73-5.64)*** |  | 720 | 51 (7.15) | 3.49 (2.24-5.43)*** |
| NMU | 498 | 12 (3.46) | 4.17 (1.78-9.77)** |  | 213 | 8 (5.21) | 2.46 (0.88-6.89) |
| Illicit drug | 37 | 5 (18.75) | 29.56 (9.77-89.42)*** |  | 41 | 7 (22.56) | 16.16 (4.94-52.84)*** |
| FTND score |  |  |  |  |  |  |  |
| Non-user | 13643 | 91 (0.78) | Ref |  | 14354 | 218 (1.73) | Ref |
| 0-3 | 2144 | 33 (1.15) | 1.78 (0.93-3.41) |  | 2695 | 86 (2.88) | 2.33 (1.55-3.48) |
| ≥4 | 2050 | 30 (1.76) | 2.60 (1.33-5.08)* |  | 1577 | 59 (4.00) | 3.19 (2.03-5.03)*** |
| AUDIT score |  |  |  |  |  |  |  |
| Non-user | 8828 | 59 (0.95) | Ref |  | 9937 | 130 (1,43) | Ref |
| 0-7 | 7932 | 76 (0.83) | 1.01 (0.61-1.66) |  | 7742 | 188 (2.61) | 2.53 (1.83-3.51) |
| ≥8 | 1077 | 19 (2.05) | 2.33 (1.05-5.15)* |  | 947 | 45 (4.17) | 4.28 (2.62-6.97)*** |
| CES-D stratum |  |  |  |  |  |  |  |
| Medium/high (29-60) | 781 | 27 (3.92) | 4.86 (2.71-8.70)*** |  | 656 | 53 (8.07) | 4.88 (3.32-7.17)*** |
| ^a^Adjusted odds ratio and its 95% confidence interval controlling for all the sociodemographic variables in Table S2.  *P<0.05; **P<0.01; ***P<0.001 | | | | | | | |

Table S3.c Correlates in other substances use and depression with past-year use of Z-drugs among participants in the 2014 and 2018 waves of the national survey.

|  | Past-year use of Z-drugs, 2014 | | |  | Past-year use of Z-drugs, 2018 | | |
| --- | --- | --- | --- | --- | --- | --- | --- |
| Variable | N | n (%_wt_) | aOR (95% CI)^a^ |  | N | n (%_wt_) | aOR (95% CI)^a^ |
| Total | 17837 | 241 (1.67) | - |  | 18626 | 205 (1.13) | - |
|  |  |  |  |  |  |  |  |
| Tobacco | 3001 | 75 (2.94) | 2.19 (1.36-3.50)** |  | 2955 | 57 (1.70) | 2.40 (1.51-3.83)*** |
| Alcohol | 7292 | 143 (2.10) | 1.78 (1.25-2.54)** |  | 7327 | 111 (1.57) | 2.71 (1.85-3.97)*** |
| Binge drinking | 587 | 17 (2.42) | 1.36 (0.69-2.68) |  | 543 | 10 (1.22) | 1.43 (0.64-3.18) |
| Areca nuts | 1141 | 35 (3.54) | 2.36 (1.32-4.21)** |  | 1247 | 23 (1.89) | 2.11 (1.12-3.98)* |
| Prescription analgesics | 1129 | 46 (3.76) | 2.27 (1.44-3.59)*** |  | 720 | 37 (5.19) | 5.12 (3.16-8.30)*** |
| NMU | 498 | 22 (4.64) | 3.10 (1.63-5.89)*** |  | 213 | 6 (1.64) | 1.36 (0.50-3.75) |
| Illicit drug | 37 | 7 (20.43) | 16.35 (6.11-43.77)*** |  | 41 | 9 (31.10) | 64.58 (23.35-178.57)*** |
| FTND score |  |  |  |  |  |  |  |
| Non-user | 13643 | 135 (1.26) | Ref |  | 14354 | 122 (0.91) | Ref |
| 0-3 | 2144 | 53 (2.25) | 1.88 (1.13-3.13) |  | 2695 | 47 (1.48) | 2.66 (1.55-4.57) |
| ≥4 | 2050 | 53 (3.27) | 2.71 (1.56-4.73)** |  | 1577 | 36 (2.33) | 4.49 (2.59-7.78)*** |
| AUDIT score |  |  |  |  |  |  |  |
| Non-user | 8828 | 59 (0.95) | Ref |  | 9937 | 76 (0.80) | Ref |
| 0-7 | 7932 | 76 (0.83) | 1.72 (1.14-2.58) |  | 7742 | 107 (1.41) | 2.31 (1.54-3.48) |
| ≥8 | 1077 | 19 (2.05) | 2.83 (1.53-5.22)** |  | 947 | 22 (1.70) | 3.08 (1.62-5.85)* |
| CES-D stratum |  |  |  |  |  |  |  |
| Medium/high (29-60) | 781 | 47 (7.16) | 5.84 (3.66-9.32)*** |  | 656 | 25 (3.56) | 3.87 (2.22-6.75)*** |
| ^a^Adjusted odds ratio and its 95% confidence interval controlling for all the sociodemographic variables in Table S2.  *P<0.05; **P<0.01; ***P<0.001 | | | | | | | |
